# Supplementary material for: Novel feature selection method via kernel tensor decomposition for improved multi-omics data analysis
Source: BMC Med Genomics. 2022 Feb 24;15:37. doi: 10.1186/s12920-022-01181-4 (PMC8876179; doi:10.1186/s12920-022-01181-4)
Supplement: Supplementary file 1 — Additional file 1. Tables S1 to S4. Supplementary Tables. [file 12920_2022_1181_MOESM1_ESM.pdf]

Table S1 Top-ranked 10 Kyoto Encyclopedia of Genes and Genomes (KEGG) pathways enriched in the Enrichr "KEGG 2019 HUMAN" category when 21 transcription factors (TFs) in Table 4 are uploaded. (The full list is available in Supplementary Data S1.)

Term	Overlap	P-value	Adjusted P-value
Signaling pathways regulating pluripotency of stem cells	6/139	4.21×10^{-8}	3.16×10^{-6}
Hippo signaling pathway	6/160	9.73×10^{-8}	3.65×10^{-6}
Pathways in cancer	8/530	6.06×10^{-7}	1.52×10^{-5}
Th17 cell differentiation	4/107	1.66×10^{-5}	3.11×10^{-4}
Hepatocellular carcinoma	4/168	9.68×10^{-5}	1.45×10^{-3}
Adherens junction	3/72	1.53×10^{-4}	1.85×10^{-3}
Pancreatic cancer	3/75	1.73×10^{-4}	1.85×10^{-3}
Colorectal cancer	3/86	2.59×10^{-4}	2.43×10^{-3}
AGE-RAGE signaling pathway in diabetic complications	3/100	4.03×10^{-4}	3.36×10^{-3}
Maturity onset diabetes of the young	2/26	6.46×10^{-4}	4.84×10^{-3}

Table S2 Top-ranked 10 Gene Expression Omnibus (GEO) profiles enriched in the Enrichr "Disease Perturbations from GEO up/down" category when 8 genes in Table 5 are uploaded (the full list is available in Supplementary Data S2).

Disease Perturbations from GEO down			
Term	Overlap	P-value	Adjusted P-value
Parkinson's disease DOID-14330 human GSE7621 sample 941	6/258	1.19×10^{-10}	5.31×10^{-8}
Teratospermia UMLS CUI-C0919628 human GSE6872 sample 953	6/372	1.08×10^{-9}	2.41×10^{-7}
autism spectrum disorder DOID-0060041 human GSE25507 sample 1029	5/297	3.77×10^{-8}	4.65×10^{-6}
asthma DOID-2841 human GSE16032 sample 729	5/303	4.17×10^{-8}	4.65×10^{-6}
familial hypercholesterolemia DOID-13810 human GSE6088 sample 908	5/317	5.22×10^{-8}	4.66×10^{-6}
relapsing-remitting multiple sclerosis DOID-2378 human GSE16461 sample 879	5/359	9.71×10^{-8}	7.22×10^{-6}
multiple sclerosis DOID-2377 human GSE16461 sample 584	5/452	3.05×10^{-7}	1.83×10^{-5}
Fracture of femur C0015802 rat GSE1685 sample 240	4/168	3.28×10^{-7}	1.83×10^{-5}
Malignant mesothelioma of pleura C0812413 human GSE2549 sample 118	4/203	6.99×10^{-7}	3.37×10^{-5}
Parkinson's disease DOID-14330 human GSE19587 sample 740	4/207	7.55×10^{-7}	3.37×10^{-5}
Disease Perturbations from GEO up			
Term	Overlap	P-value	Adjusted P-value
autism spectrum disorder DOID-0060041 human GSE25507 sample 1032	6/253	1.06×10^{-10}	5.72×10^{-8}
facioscapulohumeral muscular dystrophy DOID-11727 human GSE15090 sample 541	5/162	1.80×10^{-9}	3.19×10^{-7}
actinic keratosis DOID-8866 human GSE2503 sample 628	5/170	2.29×10^{-9}	3.19×10^{-7}
Actinic keratosis C0022602 human GSE2503 sample 350	5/171	2.36×10^{-9}	3.19×10^{-7}
melanoma in situ UMLS CUI-C0346040 human GSE4587 sample 980	5/196	4.70×10^{-9}	5.07×10^{-7}
skin squamous cell carcinoma DOID-3151 human GSE2503 sample 627	5/227	9.82×10^{-9}	8.83×10^{-7}
schizophrenia DOID-5419 human GSE21935 sample 855	5/250	1.59×10^{-8}	1.23×10^{-6}
multiple sclerosis DOID-2377 human GSE26484 sample 742	5/271	2.38×10^{-8}	1.61×10^{-6}
Schizophrenia C0036341 human GSE4036 sample 357	5/291	3.40×10^{-8}	1.87×10^{-6}
Sjogren's syndrome DOID-12894 human GSE23117 sample 889	5/292	3.46×10^{-8}	1.87×10^{-6}

Table S3 Top-ranked 10 Gene Expression Omnibus (GEO) profiles enriched in the Enrichr "Disease Perturbations from GEO up/down" category when proteins listed in the WBC section of Table 6 are uploaded (the full list is available in Supplementary Data S3).

Disease Perturbations from GEO down			
Term	Overlap	P-value	Adjusted P-value
ulcerative colitis DOID-8577 human GSE11223 sample 593	11/297	1.35×10^{-14}	4.99×10^{-12}
autism spectrum disorder DOID-0060041 human GSE7329 sample 1034	11/308	2.02×10^{-14}	4.99×10^{-12}
psoriasis DOID-8893 human GSE14905 sample 754	8/322	2.43×10^{-9}	4.01×10^{-7}
asthma DOID-2841 human GSE16032 sample 729	7/303	4.74×10^{-8}	5.86×10^{-6}
Non-syndromic cleft lip and palate DOID-9296 human GSE42589 sample 618	7/426	4.79×10^{-7}	4.73×10^{-5}
autistic disorder DOID-12849 human GSE6575 sample 1042	5/143	6.64×10^{-7}	5.46×10^{-5}
hepatitis C DOID-1883 human GSE20948 sample 600	6/307	1.33×10^{-6}	9.37×10^{-5}
Hereditary gingival fibromatosis C0399440 human GSE4250 sample 177	6/321	1.72×10^{-6}	9.97×10^{-5}
cystic fibrosis DOID-1485 mouse GSE33319 sample 1048	6/324	1.82×10^{-6}	9.97×10^{-5}
Huntington's disease DOID-12858 human GSE1751 sample 795	5/213	4.71×10^{-6}	2.33×10^{-4}
Disease Perturbations from GEO up			
Term	Overlap	P-value	Adjusted P-value
systemic juvenile idiopathic arthritis (sJIA) DOID-848 human GSE21521 sample 574	11/367	1.38×10^{-13}	3.91×10^{-11}
acute myeloid leukemia DOID-9119 human GSE9476 sample 782	9/163	1.49×10^{-13}	3.91×10^{-11}
Down syndrome DOID-14250 human GSE19681 sample 1066	10/280	4.06×10^{-13}	7.08×10^{-11}
H1N1 DOID-0050211 human GSE27131 sample 514	11/427	7.19×10^{-13}	9.40×10^{-11}
hepatocellular carcinoma DOID-684 human GSE58208 sample 735	10/455	4.96×10^{-11}	5.19×10^{-9}
Lewy body dementia DOID-12217 human GSE49036 sample 1068	9/366	2.14×10^{-10}	1.87×10^{-8}
Schistosomiases C0036323 mouse GSE19525 sample 439	9/485	2.55×10^{-9}	1.90×10^{-7}
Huntington's disease DOID-12858 human GSE8762 sample 931	7/315	6.19×10^{-8}	3.76×10^{-6}
polycystic ovary syndrome DOID-11612 human GSE10946 sample 825	7/320	6.89×10^{-8}	3.76×10^{-6}
systemic juvenile idiopathic arthritis (sJIA) (enthesitis-related) DOID-848 human GSE21521 sample 575	7/322	7.19×10^{-8}	3.76×10^{-6}

Table S4 Top-ranked 10 Gene Expression Omnibus (GEO) profiles enriched in the Enrichr "Disease Perturbations from GEO up/down" category when proteins listed in the plasma section of Table 6 are uploaded (the full list is available in Supplementary Data S4).

Disease Perturbations from GEO down			
Term	Overlap	P-value	Adjusted P-value
Nemaline Myopathy C0206157 mouse GSE3384 sample 160	12/324	1.49×10^{-16}	5.81×10^{-14}
nemaline myopathy DOID-3191 mouse GSE3384 sample 976	12/450	7.68×10^{-15}	1.50×10^{-12}
nemaline myopathy DOID-3191 mouse GSE3384 sample 975	10/239	2.80×10^{-14}	3.64×10^{-12}
Hepatitis, Autoimmune C0241910 mouse GSE867 sample 230	8/227	6.79×10^{-11}	6.62×10^{-9}
Carcinoma, Hepatocellular C0019204 human GSE6764 sample 407	8/303	6.73×10^{-10}	5.25×10^{-8}
Gastrointestinal stromal tumor C0238198 human GSE2719 sample 270	6/224	1.19×10^{-7}	7.71×10^{-6}
atherosclerosis DOID-1936 mouse GSE19286 sample 906	6/259	2.79×10^{-7}	1.55×10^{-5}
Type 2 diabetes mellitus C0011860 mouse GSE2899 sample 45	6/268	3.41×10^{-7}	1.66×10^{-5}
Hepatocellular carcinoma DOID-684 human GSE10393 sample 493	6/298	6.35×10^{-7}	2.48×10^{-5}
Carcinoma, Hepatocellular C0019204 mouse GSE2127 sample 300	6/298	6.35×10^{-7}	2.48×10^{-5}
Disease Perturbations from GEO up			
Term	Overlap	P-value	Adjusted P-value
intracranial aneurysm DOID-10941 human GSE26969 sample 744	13/163	2.01×10^{-22}	8.89×10^{-20}
Hyperlipidemia C0020473 rat GSE3512 sample 38	12/277	2.24×10^{-17}	4.97×10^{-15}
Hepatic Cirrhosis C0023890 rat GSE1843 sample 372	12/297	5.21×10^{-17}	7.70×10^{-15}
Sepsis C0243026 rat GSE1781 sample 314	10/226	1.60×10^{-14}	1.77×10^{-12}
NASH C0400966 human GSE24807 sample 185	10/275	1.15×10^{-13}	1.02×10^{-11}
Burn C0006434 rat GSE802 sample 97	9/283	8.48×10^{-12}	6.26×10^{-10}
Hepatic lipidosis C0015695 mouse GSE5538 sample 10	9/316	2.27×10^{-11}	1.44×10^{-9}
Vitamin A Deficiency C0042842 rat GSE1600 sample 359	8/265	2.33×10^{-10}	1.29×10^{-8}
Atherosclerosis C0004153 mouse GSE363 sample 193	8/299	6.06×10^{-10}	2.98×10^{-8}
colitis DOID-0060180 mouse GSE34874 sample 800	8/345	1.87×10^{-9}	8.30×10^{-8}
